# On the Respiratory Function in Pneumonia

**Published:** 1866-03

**Authors:** J. P. Ross

**Affiliations:** Attending Physician to the County Hospital


*On the Respiratory Function in Pneumonia: Report of Cases.* By Dr. J. P. Ross, Attending Physician to the County Hospital.

The two following cases of Pneumonia are reported, not because there is anything very remarkable in them, but to show the importance of keeping up the respiratory function especially where a large portion of the lungs are involved in the disease; and secondly, the danger which may attend the administration of opium, from its effects in diminishing the respirations.

CASE I. DOUBLE-PNEUMONIA.

R. S. W——d, aged 23, brakeman on a railroad, sent for me, May 1st, 1859. He said, he was taken four days before with a chill, followed by headache, loss of appetite, occasional cough, and a dull pain in the right breast. A general examination revealed the skin moist; pulse, 100, full; tongue but slightly furred; bowels free from the action of a cathartic which had been administered the day before; there was no appetite and but little thirst. The patient was sitting up in bed, making but little complaint. On percussion, there was dullness over two-thirds of the right lung inferiorly, with bronchial respiration. Vocal resonance increased. There was slighter dullness and crepitation over left back inferiorly. The sputum was copious and rust-colored. The respirations were twenty per minute. Prescribed three grains of quinine and one-third of a grain of morphine every fourth hour.

May 2d. Patient very much as yesterday. In addition to the physical signs, dullness was found over lower and anterior portion of left lung, also bronchial respiration. The respirations were 21 per minute and somewhat labored. Urine scanty and high colored. Warm fomentations were ordered to the chest; the powders to be continued.

May 3d, 8 o'clock A. M. Was called in great haste, as patient was reported to be dying. His face was of a bronzed hue; the respirations were labored and catching; pulse could scarcely be felt, small and frequent; lips blue; the body was slightly convulsed; extremities cold, and the patient unconscious. The respiratory murmur was absent, with dullness on percussion over two-thirds of right and left lungs inferiorly; also, coarse crepitation over remaining portion. Ordered to be given immediately one ounce of brandy, hot bottles to the extremities, and hot fomentations to the chest, and wrote for the following:

℞ Ammon Carb.,

Camph. pulv., aa ʒss.

Quiniæ sulph., gr. x. M. Ft. ch. No. x.

S. One powder to be given as soon as obtained, and to be repeated every third hour. And with the first and every other powder, to give one teaspoonful of the following:

| | | |
|---|---|---|
| ℞ Strychinæ, | gr. i. | |
| Acid nitric, dil., | ʒi. | |
| Aquæ, | ℥ii. | M. |

At 3 o'clock P. M., symptoms greatly improved; respirations 39 per minute, less labored and regular; pulse, 110, full; intellect clear; anxiety of countenance less; complexion florid.

May 4th. Patient rested well last night; doing well. Continue treatment.

May 5th. Crepitation is fully established over upper and middle portion of both lungs; skin moist; pulse, 91; bowels not moved for three days. Continue treatment, and in addition give three com. cath. pills at bedtime.

May 6th. Bowels moved; appetite returning; ate a piece of beafsteak for breakfast; chlorides in abundance in the urine, which was not tested before to-day. The strychnine mixture to be discontinued, and the powders to be given every fourth hour.

May 7th. Found patient dressed and sitting up. The powder to be discontinued.

May 8th. The respiratory murmur normal over both lungs with but slight crepitation; patient dismissed, cured.

*Remarks.*—On my first visit, the inferior lobe of the right lung seemed to have progressed to the stage of hepatization, and the disease was spreading in the left lung, which became extensively involved by the morning of the third day. At this time probably two-thirds of both lungs were entirely disabled for the performance of their function. And in addition, there was congestion of the remaining portion of sound lung from venous blood seeking to be arterialized; and secondly, there was venous blood, narcotizing the brain and thus suspending the respiratory function, which afforded the only means of relief. Now what was the indication of treatment? Evidently to stimulate the respirations. For this purpose, stimulants were selected which would act more especially on the brain and nervous system. The success afforded in re-establishing the respirations and pulse, is revealed in the preceding history. After two doses of the remedies were administered, the respirations were thirty per minute; pulse, 110, full; intellect clear, and complexion florid. Nothing more was done but a continued use of the remedies to keep the patient breathing, and the recovery was most rapid.

How much the opium in the first prescription contributed to the alarming symptoms which occurred on the morning of the third day, I am unable to say; but I believe it had something to do with it; and my subsequent experience has taught me to be cautious in the use of this remedy in many cases of pneumonia. In all such cases, as above, where venous blood mingled in the general circulation, I believe opium to be a dangerous remedy.

CASE II.

Wm. B——, American; aged 60; laborer of light work in a bakery; sent for me, Nov. 20th, 1863. His wife states that he was attacked, three days before, with chills, alternating with fever; slight cough; bloody sputa; restless, and at times delirious. Had taken a cathartic and several 3 gr. powders of quinine, but continued to grow worse to the present time. On examination, the skin was hot and dry; pulse, 116, full; tongue coated with thin, white fur; slight thirst; no appetite; delirious. Physical exploration revealed dullness, bronchial respiration and broncophony over the larger portion of the right lung. Ordered five grains of Dover powder, with $\frac{1}{10}$th of grain of tartar emetic, every four hours, and warm fomentations over the affected side.

Nov. 21st. Patient worse; very restless and delirious; slept but little since attacked. Ordered $\frac{1}{3}$ gr. morphine and 5 grs. camphor at bedtime, and the same quantity repeated every two hours until sleep is induced.

Nov. 22d. 6 o'clock A. M. Patient in a deplorable state. Countenance and prolabia blue; unconscious; breathing irregular and jerking; pulse hardly perceptible at the wrist; deglutition impossible. No sleep had been induced: five of the powders had been taken. Ordered hot turpentine stupes to chest and abdomen, mustard to the feet and legs, and administered ten grains of quinine and four ounces of whisky per rectum. In twenty minutes patient able to swallow, with some difficulty, an occasional teaspoonful of whisky and water. Wrote for the following: Carb. ammoniæ and pulv. camphor, each 30 grains; quinine, 10 grains,—made into ten powders, one to be given every two hours. With the first, and every other powder, to give one teaspoonful of a solution containing $\frac{1}{16}$th gr. of strychnia.

1 o'clock P. M. Capillary circulation, pulse and respirations much improved. Two powders and one dose of the strychnia had been administered. Ordered an interval of three hours to elapse between the powders, and six hours between the doses of strychnia. Beef-tea and milk-punch every hour alternately.

Nov. 23d. Delirium continues; pulse and respirations much as when last visited. Bronchial respiration and dullness over whole of right lung; continue treatment.

Nov. 24th. General symptoms improved. Crepitation returning over affected side; expectoration more copious.

Nov. 25th. Patient rational; bowels relaxed with pain. Omit the powders, and continue the strychnia mixture with ten drops of tinct. opii with each dose. From this date convalescence progressed steadily and rapidly. On the 28th, medical treatment was discontinued, and the patient dismissed, cured.

*Remarks.*—The alarming symptoms which presented on the morning of the 22d threatened death, as in the preceding case, by asphyxia. This was revealed by the blueness of the prolabia and skin, and the irregular and jerking respirations; and the indications for treatment were precisely the same in both cases, namely, to stimulate the respirations.

It is stated by eminent authority that pneumonia kills by asthenia or exhaustion and not by apnœa. This is plainly stated in a recently published clinical lecture on pneumonia by Dr. Flint. From my own observations I am induced to believe this rule (if such it is) is subject to a great many exceptions, of which the above are cases. Indeed, I believe one of the great dangers of this disease is the failure of the lungs to sufficiently decarbonize and oxygenate the blood. Nature hangs out the sign of this want in the bronzed or mahogany hue of the complexion. Hence, attention to the respiratory function should particularly engage the attention of the medical practitioner throughout the whole course of the disease. Recognizing the liability to asphyxia, it follows that great care should be had in administering opium, especially where a large portion of the lung tissue is disabled by the disease; for opium, by lessening the frequency of the respirations, and the disease lessening the oxygenating surface, the remedy and disease combine to precipitate the patient into an alarming state of asphyxia. This I conceive to have been the result in the cases reported above, and my experience induces me to be more cautious in the use of this drug. Nevertheless, the treatment of pneumonia with large doses of opium has good authority to recommend it. But if this mode of treatment has either science or experience to recommend it, I have failed to make the discovery.

